# *Helicobacter pylori* Infection Acts Synergistically with a High-Fat Diet in the Development of a Proinflammatory and Potentially Proatherogenic Endothelial Cell Environment in an Experimental Model

**DOI:** 10.3390/ijms22073394

**Published:** 2021-03-25

**Authors:** Agnieszka Krupa, Weronika Gonciarz, Paulina Rusek-Wala, Tomasz Rechciński, Adrian Gajewski, Zuzanna Samsel, Anna Dziuba, Agnieszka Śmiech, Magdalena Chmiela

**Affiliations:** 1 Institute of Microbiology, Biotechnology and Immunology, Department of Immunology and Infectious Biology, Faculty of Biology and Environmental Protection, University of Lodz, Banacha st 12/16, 90-237 Lodz, Poland; weronika.gonciarz@biol.uni.lodz.pl (W.G.); paulina.rusek.wala@edu.uni.lodz.pl (P.R.-W.); or zuzanna.kalista@gmail.com (Z.S.); ania1995ania@gmail.com (A.D.); agnieszka.smiech@biol.uni.lodz.pl (A.Ś.); magdalena.chmiela@biol.uni.lodz.pl (M.C.); 2 The Bio-Med-Chem Doctoral School, University of Lodz and Lodz Institutes of the Polish Academy of Sciences, Banacha st 12/16, 90-237 Lodz, Poland; 3 Clinic and Department of Cardiology, Medical University of Lodz, 92-213 Lodz, Poland; rechcinski@gmail.com; 4 Department of Immunology and Allergy, Medical University of Lodz, Pomorska 251, 92-213 Lodz, Poland; adrian.gajewski@umed.lodz.pl

**Keywords:** *Helicobacter pylori*, endothelium, atherosclerosis, inflammation

## Abstract

Classic atherosclerosis risk factors do not explain all cases of chronic heart disease. There is significant evidence that gut microbiota may influence the development of atherosclerosis. The widespread prevalence of chronic *Helicobacter pylori* (*H. pylori*, *HP*) infections suggests that *HP* can be the source of components that stimulate local and systemic inflammatory responses. Elevated production of reactive oxygen species during *HP* infection leads to cholesterol oxidation, which drives atherogenesis. The aim of this study is to explore the link between persistent *HP* infection and a high-fat diet in the development of proinflammatory conditions that are potentially proatherogenic. An in vivo model of *Caviae porcellus* infected with *HP* and exposed to an experimental diet was investigated for the occurrence of a proinflammatory and proatherogenic endothelial environment. Vascular endothelial primary cells exposed to *HP* components were tested in vitro for oxidative stress, cell activation and apoptosis. The infiltration of inflammatory cells into the vascular endothelium of animals infected with *HP* and exposed to a high-fat diet was observed in conjunction with an increased level of inflammatory markers systemically. The arteries of such animals were the least elastic, suggesting the role of *HP* in arterial stiffness. Soluble *HP* components induced transformation of macrophages to foam cells in vitro and influenced the endothelial life span, which was correlated with Collagen I upregulation. These preliminary results support the hypothesis that *HP* antigens act synergistically with a high-fat diet in the development of proatherogenic conditions.

## 1. Introduction

Coronary heart disease (CHD) is one of the most severe chronic diseases of the coronary vessels, and an important health and social problem that is often life-threatening. Classic risk factors of CHD include cigarette smoking, hypertension, diabetes mellitus and elevated levels of triglycerides and total cholesterol, especially the fraction of low-density lipoprotein (LDL) [1,2]. CHD develops due to endothelial dysfunction within the vessels, especially in the presence of elevated cholesterol and inflammation [3], which results in increased blood pressure, vascular wall remodeling, vascular inflammation development, increased platelet adhesion and aggregation. Such disorders promote the formation of atheromatous plaques, which are often unstable and subsequently rupture [2]. This might impair the blood flow leading to vascular blockage or myocardial infarction [1]. Importantly, classic risk factors for development of atherosclerosis do not explain all cases of CHD; therefore, the concept that atherogenesis may have an infectious background is extremely possible. Chronic infections may influence the course of CHD via different mechanisms, such as chronic inflammatory reactions, autoimmune processes, and modification of the classic risk factors of CHD [4,5]. There is substantial evidence that gut microbiota, particularly *Escherichia coli* lipopolysaccharide leaking from the gut, may influence the development of atherosclerosis via Toll-like receptor 4 (TLR-4)-mediated oxidative stress [6,7]. The role of *Helicobacter pylori* (*H. pylori, HP*) infection in the development of CHD was suggested for the first time by Mendall in 1994. He showed the elevation of anti-*H. pylori* antibodies in the sera of patients suffering from CHD [8]. Even though other authors have confirmed this serological observation [9,10,11,12], the evidence of these bacteria’s influence on the development of atherosclerosis is still not definitive.

The unique mechanism of *H. pylori* invasion and survival in the organism is mostly based on the bacterial ability to colonize gastric epithelial cells, via the direct action of the soluble bacterial components or adhesins facilitating binding of bacterial cells with epithelial cell receptors. Furthermore *H. pylori* can modulate immunocompetent cells’ activity [13,14,15,16,17]. In the acute phase of infection, *H. pylori* induces an excessive inflammatory response in the gastric mucosa, which is accompanied by the release of oxidative stress molecules, such as reactive oxygen species (ROS), and various soluble or cellular compounds—products of bacterial cell lysis [18]. Excessive inflammation can cause gastric epithelial barrier impairment and a deficiency in its protective function. Moreover, the loss of epithelial barrier integrity can facilitate the translocation of soluble *H. pylori* virulence factors into the circulation. Strong vascularization of the gastric area allows *H. pylori* components to interact with fibroblasts, endothelial cells, and immunocompetent cells [19,20,21,22]. The last ones should contribute to the elimination of the infection and propagation of damaged tissue reparation; however, an excessive activation of host cells may promote the development of chronic inflammation in conjunction with induction of pathological processes [23].

Soluble antigenic compounds of *H. pylori* may affect vascular endothelium directly by interactions with it, indirectly via form bound with leukocytes or due to lipid oxidation, which provides oxidized LDL (oxLDL) fractions—classic risk factors of CHD [24,25]. Due to the high concentration of ROS accompanying *H. pylori* infection, cholesterol may undergo oxidation to proatherogenic 7-KCh, which drives atherogenesis [21,22]. Furthermore, *H. pylori* components delivered to the circulation may influence immunocompetent cells, including monocytes, to transform into foam cells, which are involved next in the development of atherosclerotic plaque. This process is correlated with lipid deposition in these cells. Both *H. pylori* proteins and lipopolysaccharide (LPS) demonstrate proinflammatory properties. We have shown previously [26] the increased permeability of cellular monolayers of gastric epithelial cells in the milieu of *H. pylori* components [26]. This could be due to increased oxidative stress and upregulation of epithelial cell apoptosis [21,22]. Our preliminary study indicated also that endothelial cells exposed to *H. pylori* antigenic components became activated via phosphorylated extracellular signal-regulated kinase (pERK) signaling pathway [27].

The widespread prevalence of *H. pylori* infections and the fact that they are frequently asymptomatic may suggest that, similarly to intestinal microflora, *H. pylori* can be a source of antigenic components that stimulate not only local but also systemic inflammatory response [5]. Considering the mechanism of *H. pylori* pathogenicity, which results in massive oxidative stress induction and gastric epithelial barrier disintegration, it is possible that the *H. pylori* soluble components translocated into the circulation may act synergistically with a high-fat diet in the development of a proinflammatory and proatherogenic endothelial cell environment.

## 2. Results

### 2.1. Validation of H. pylori Infection in an Experimental In Vivo Model of Atherosclerosis

To explore the link between persistent *H. pylori* infection and a high-fat diet in the development of atherosclerosis, we employed an in vivo model of *Caviae porcellus* infected with *H. pylori* and exposed to an experimental diet. The model of *H. pylori* infection in guinea pigs is useful due to induction of the inflammatory and immune responses that is analogous to humans [28,29,30]. Thus, guinea pigs respond to infection with both a humoral and cellular immune response and by elevation of acute-phase proteins [29,30,31,32]. A study done in our scientific team by Walencka et al. [30] described the condition of animal inoculation with *H. pylori* eligible to develop the inflammatory response locally in the gastric mucosa and systemically (7 and 28 days of infection, respectively). We extended the infection time to 60 days and introduced animals to an experimental high-fat diet to include the dietary risk factor of CHD to the experimental setup [33,34]. Animals uninfected and inoculated with *H. pylori* exposed to the normal chow diet served as the control.

To confirm the colonization of *H. pylori* in the gastric tissue of inoculated vs. non-exposed animals, sections stained with Giemsa and H&E methods were analyzed according to several principles of the Sydney system, which was a classification of gastritis introduced in 1990 and updated in 1995 [30,35,36]. Histopathological verification was done by two independent histopathologists and based on microscopic observation of *Helicobacter*-like organisms (HLO). The visual grading system for HLO was applied as follows: 0—no bacteria detected in gastric crypts; 1—mild level of colonization (some bacteria detected in gastric crypts); 2—moderate level of colonization (bacteria detected in most gastric crypts); and 3—severe level of colonization (bacteria present in all gastric crypts). As shown in Table 1, the level of colonization in infected animals, regardless of diet, at 7 days of infection was at grade 1, whereas at 28 and 60 days of infection at a grade in the range of 1–2. HLO were not detected in the gastric tissue of the control animals inoculated with complete *Brucella* broth (grade 0).

**Figure 1:**
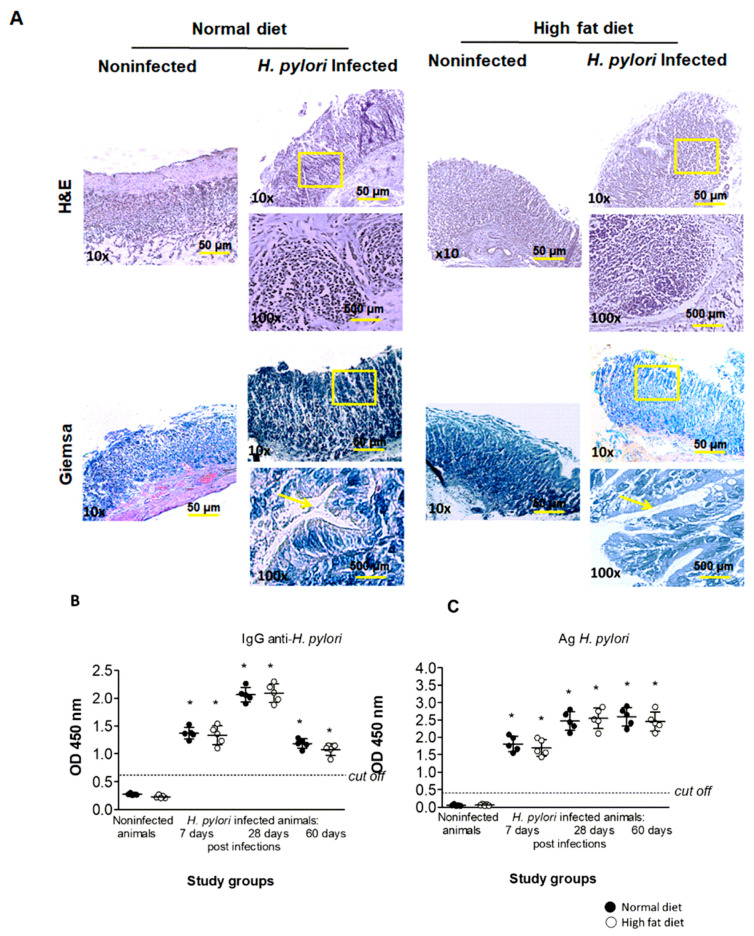
Histological and serological assessment of *H. pylori* infection in the experimental guinea pig model. (**A**) Representative images of the Giemsa- and H&E-stained thin layer sections of gastric tissue from uninfected or *H. pylori*-infected animals after 60 days from inoculation. Uninfected or *H. pylori*-infected animals were exposed to normal chow (left panel) or an experimental high-fat diet (right panel). Stained sections were analyzed using light microscopy (magnification ×100, ×400). The yellow arrows show the location of *Helicobacter*-like organisms (HLO). (**B**) The serum level of anti-*H. pylori* IgG and (**C**) the detection of *H. pylori* antigens (Ag) in stool samples of uninfected and *H. pylori*-infected guinea pigs after 7, 28 and 60 days from inoculation. Five animals per group were examined. The results are presented as the median values of four independent experiments performed in triplicates for each experimental variant. Statistical analysis was performed with the nonparametric Kruskal–Wallis test. Statistical significance, * *p* < 0.05, was obtained for *H. pylori*-infected animals (7, 28 and 60 days after inoculation) fed chow or a high-fat diet vs. the control animal.

The typical localization of HLO in gastric tissue of infected animals at 60 days of infection is shown on the representative pictures in Figure 1A (yellow arrows point the localization of the *Helicobacter*-like organisms—HLO). Assessment of the *H. pylori* status by culture was not done, because the series of studies done in our laboratory [30] revealed the existence of mixed flora in the guinea pig stomach, which excludes the test as specific. Moreover, we tested the presence of *H. pylori* antigens in the stool samples and anti-*H. pylori* specific antibodies in the serum of animals of all groups. The concentration of *H. pylori* antigens in stool samples is considered a diagnostic tool for confirmation of *H. pylori* colonization and it was performed as previously described by Walencka et al. 2015 [30]. As shown in Figure 1C, the concentration of *H. pylori* antigens in stool samples was higher at 28 and 60 days in comparison to 7 days from inoculation. The colonization of animals with *H. pylori* results in the production of specific anti-*H. pylori* antibodies of IgM and IgG isotype, which is also a common reaction to this infection in humans [21]. Our results confirm the persistence of *H. pylori* infection because the level of anti-*H. pylori* IgG in serum of all infected animals, regardless of diet, reached the maximum after 28 days from inoculation. Interestingly, at the time of 60 days from inoculation, the level of anti-*H. pylori* IgG dropped to the point detected in animals at 7 days of infection (Figure 1B). It is probable that anti-*H. pylori* IgG can be either removed or trapped in the immune complexes. The presence of such complexes was detectable in the serum of patients infected with *H. pylori* and suffering CHD [37].

Histological analysis of the gastric tissue of animals of all groups also included a verification of inflammatory cell (granulocytes and lymphocytes) infiltration. The grading system for such an analysis was applied as follow: 0—no infiltration of inflammatory cells; 1—moderate infiltration of inflammatory cells; and 2—increased infiltration of inflammatory cells (as described previously by Walencka et al. [30]). Our results presented in Table 1 show that the inflammatory cell infiltration in gastric tissue of infected animals, regardless of diet, was graded 1 at 7 days of infection and in the range of 1–2 at 28 and 60 days of infection. There was no evidence of infiltration of inflammatory cells (grade 0) in the gastric tissue of control animals inoculated with *Brucella* broth only.

**Figure 2:**
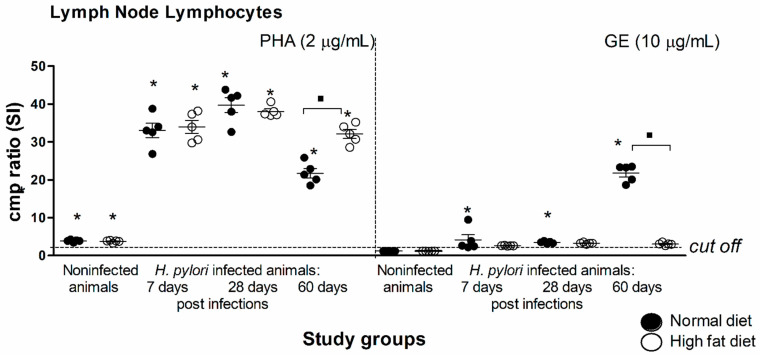
*H. pylori* antigen-driven proliferative response of lymph node lymphocytes in the single-stage cultures of total mesenteric lymph node leukocytes (tMLNL) incubated for 72 h with an *H. pylori* antigenic complex—glycine acid extract (GE) or phytohemagglutinin (PHA). The proliferating activity of the lymphocytes was evaluated based on (3*H*)-thymidine incorporation. The stimulating index (SI) was calculated by dividing the radioactivity counts (cpm) for the cell cultures in the presence of the stimulus by the counts for control cell cultures in RPMI-1640 alone. Cells from 5 animals per group were examined. The results are shown as the SI ± SD. Statistical analysis was performed with the nonparametric Kruskal–Wallis test. Statistical significance, * *p* < 0.05 was obtained for *H. pylori*-infected animals fed a normal chow or a high-fat diet vs. the control animals, ▪ *p* < 0.05 was obtained for *H. pylori*-infected animals fed normal chow vs. fed a high-fat diet.

Finally, we detected the *H. pylori* antigen-driven proliferative response of the lymph node lymphocytes from *H. pylori*-infected animals exposed to normal chow or a high-fat diet, as described previously [21,38]. As shown in Figure 2, lymphocytes from animals infected with *H. pylori* and fed normal chow or a high-fat diet proliferated effectively in response to phytohemagglutinin (PHA), which indicated the increased nonspecific polyclonal activation. Interestingly, lymphocytes isolated from the lymph nodes of infected animals at 60 days of infection, but fed normal chow, proliferated very effectively towards *H. pylori* glycine extract (GE), suggesting development of an antigen-specific local response (Figure 2). On the other hand, the exposure of infected animals to a high-fat diet caused inhibition of lymph node lymphocyte antigen-specific proliferation, as shown in Figure 2**.** Our results suggest that introducing infected animals to a high-fat diet led to lymph node lymphocyte unresponsiveness to stimulation with *H. pylori* antigens. Thus, lymphocytes showed insensitivity to *H. pylori* antigens even though these bacteria were still detectable in the gastric tissue of infected animals (Table 1, Figure 1A,C).

### 2.2. Inflammatory Process in Experimental Atherosclerosis Developed in Caviae porcellus Colonized with H. pylori

**Figure 3:**
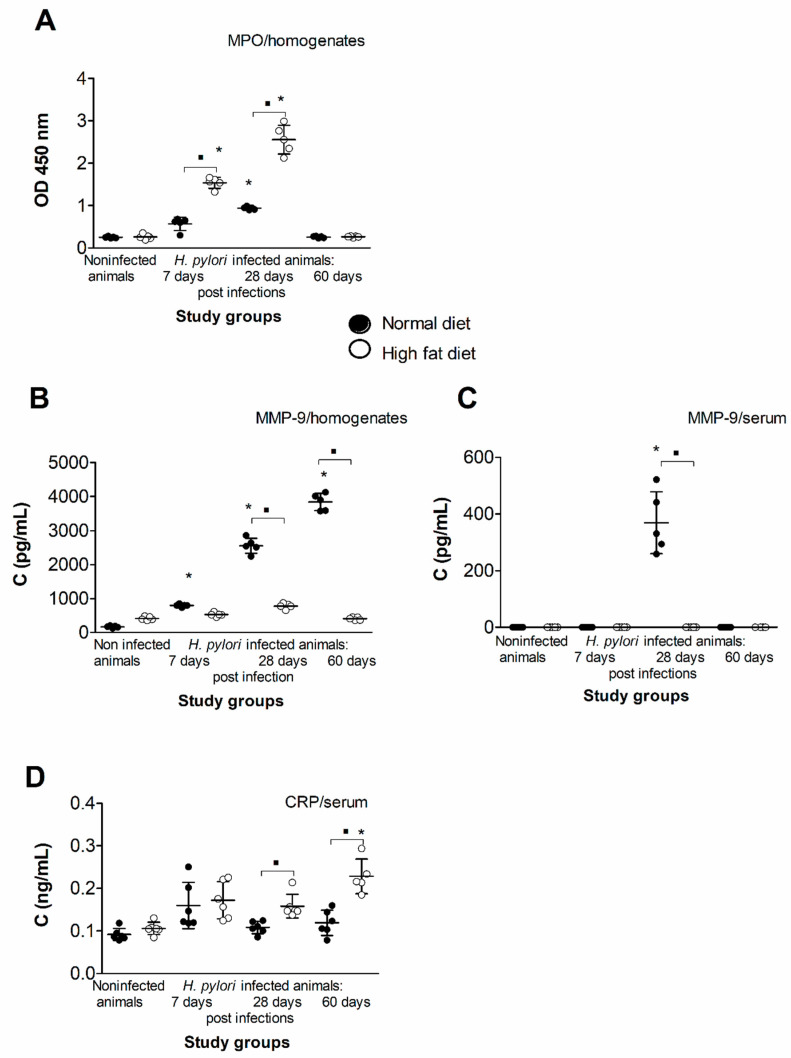
Detection of myeloperoxidase (MPO) (**A**) and metalloproteinase (MMP)-9 (**B**) in the gastric tissue and the level of MMP-9 (**C**) and C-reactive protein (CRP) (**D**) in the serum of uninfected or *H. pylori*-infected guinea pigs at 7, 28 and 60 days from inoculation. Animals were exposed to normal chow or an experimental high-fat diet. Five animals per group were examined. The results are presented as the median values. Statistical analysis was performed with the nonparametric Kruskal–Wallis test. Statistical significance, * *p* < 0.05 was obtained for *H. pylori*-infected animals fed a normal chow or a high-fat diet vs. the control animals, ▪ *p* < 0.05 was obtained for *H. pylori*-infected animals fed a normal chow vs. high-fat diet.

We evaluated the level of myeloperoxidase (MPO) and metalloproteinase (MMP)-9 in the gastric tissue of animals of all groups. Both are recognized markers of oxidative stress and inflammatory response and were shown previously by Gonciarz et al. 2019 [21] to be elevated in gastric tissue of *H. pylori*-infected guinea pigs. Our results presented in Figure 3A indicate that the concentration of MPO in gastric homogenates of infected animals reached the maximum at 28 days of infection, but it was on the level of control at 60 days from inoculation. Interestingly, the exposure of infected animals to high-fat diet raised the MPO level almost two times (Figure 3A). We also measured the level of MMP-9 in gastric tissue and serum of animals of all groups. The data shown in Figure 3B indicate that the concentration of MMP-9 in gastric tissue continuously increased over time of *H. pylori* infection; thus, at 60 days from inoculation, it was almost two times higher than at 28 days. Interestingly, the exposure of infected animals to the experimental high-fat diet caused significant downregulation of MMP-9 release, so the concentration of this enzyme at each time point reached the level of the control. Similar results were obtained for the MMP-9 in the serum of animals of all groups. As shown in Figure 3B, MMP-9 was measurable only in the group of *H. pylori*-infected animals fed normal chow at 28 days from inoculation.

Finally, in the serum of animals of all groups we measured the concentration of C-reactive protein (CRP), the marker of inflammation, which is considered the indicator of coronary events associated with endothelial damage. As shown in Figure 3D, the concentration of CRP in the serum of animals infected with *H. pylori* (60 days from inoculation) and exposed to the experimental diet was significantly higher in comparison to the other groups.

### 2.3. Diminished Vascular Elasticity in Caviae porcellus Colonized with H. pylori in Conjunction with a High-Fat Diet

**Figure 4:**
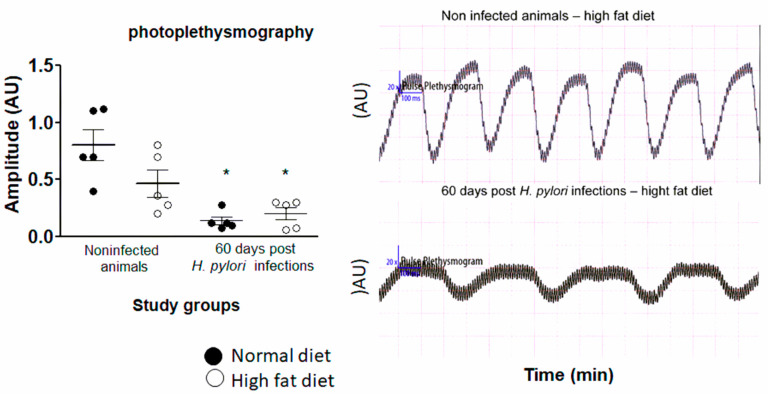
Photoplethysmography analysis of the arteries of uninfected and *H. pylori*-infected animals, at 60 days from inoculation, exposed to normal chow or an experimental high-fat diet. Five animals per group were examined. The results are presented as the mean values of the amplitudes (AU) ± SD. Statistical analysis was performed with the nonparametric Kruskal–Wallis test. Statistical significance, * *p* < 0.05, was obtained for *H. pylori*-infected animals vs. the control animals.

Vascular elasticity is an important marker that may help to measure the effects of atherosclerosis processes. To study vascular elasticity in *Caviae porcellus* colonized with *H. pylori* and exposed to a high-fat diet, we employed a special Rodent Surgical Monitor with a high-resolution electrocardiogram—ECG (Indus, Animalab, Poznan, Poland). The apparatus estimates the arteries’ stiffness by detecting the amplitude of the pulse wave. The correlation between the arteries’ stiffness and amplitude is as follows: the stiffer the artery, the lower the amplitude of the pulse wave. The measurement was done on animals of all groups at 60 days of infection. Results presented in Figure 4 indicate that the amplitude of the pulse wave was the lowest for animals infected with *H. pylori*, regardless of the presence of high-fat substances in the experimental setup. On the other hand, the amplitude of the pulse wave for animals uninfected but exposed to a high-fat diet was only slightly lowered than for normal animals. Interestingly, our data indicate (Figure 4) that the bacterial components of *H. pylori* even without accompaniment of a high-fat diet may be essential for diminishing the elasticity of arteries. We showed previously that this could be potentially due to the deposition of the immune complexes [37]. When both risk factors for atherosclerosis development, the infectious and dietary components, coexist, they can act synergistically, as presented in Figure 4.

**Figure 5:**
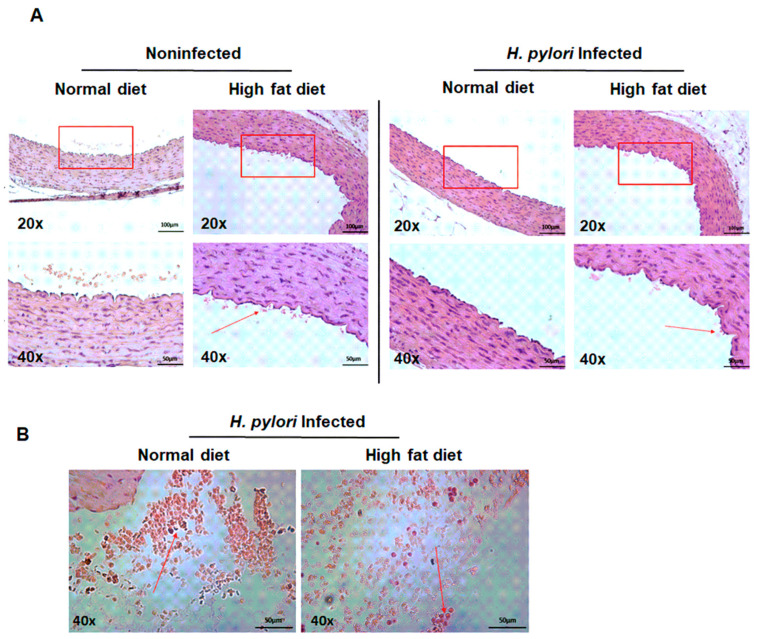
Histological analysis of aortic structure of uninfected and *H. pylori*-infected animals, at 60 days from inoculation, exposed to normal chow or an experimental high-fat diet. Representative images of H&E-stained thin layers of aortic tissue, visualizing (**A**) the aortic wall layers or (**B**) the aortic lumen. Stained sections were analyzed with light microscopy at magnifications of 200× and 400×. The red arrows show the location of the immunocompetent cells and erythrocytes in the nearest proximity to the aortic wall (**A**) or leukocyte cells in the lumen of the aorta (**B**). Five animals per group were examined.

To further evaluate the role of the *H. pylori* components and a high-fat diet in creating a proatherogenic endothelial environment, we performed a histological evaluation of the H&E-stained thin layer sections of aortic tissue of animals of all experimental groups. The histological analysis showed that none of the animals from the *H. pylori*-infected groups (at 60 days from inoculation) developed typical atherosclerotic plaques in their aortic vessels, even in the presence of fatty substances (Figure 5A). However, we observed some immunocompetent cells in the lumen of aortic vessels of animals infected with *H. pylori* and exposed to a high-fat diet (Figure 5B); moreover, some leukocytes were adhering to the aortic wall of this animals, which may indicate the generation of inflammatory conditions (Figure 5A).

### 2.4. Established Role of H. pylori Components in Transformation of Macrophages into Foam Cells

**Figure 6:**
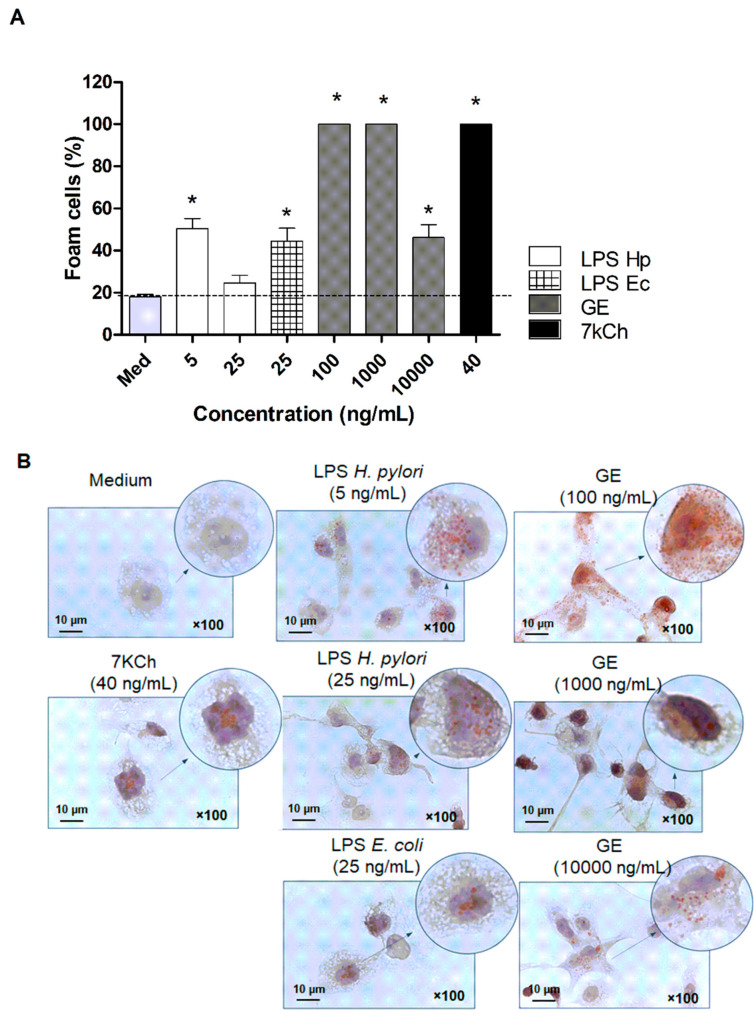
Transformation of THP-1 macrophages into foam cells. (**A**) Percentage of foam cells developed from THP-1 macrophages treated with an *H. pylori* antigenic complex—glycine acid extract (GE), lipopolysaccharide of *H. pylori* (LPS Hp) and *E. coli* (LPS Ec), and 7 ketocholesterol (7 kCh). Results are presented as the mean values ± SD. Statistical analysis was performed with the nonparametric Kruskal–Wallis test. Statistical significance, * *p* < 0.05, was obtained for THP-1 macrophages treated with the stimulators vs. control cells. (**B**) Representative pictures of the foam cells analyzed with light microscopy at a 1000× magnification.

To study the contribution of *H. pylori* components to transform macrophages into foam cells, we employed an in vitro model of THP-1 cells that changed into macrophages with phorbol myristate acetate (PMA) treatment. THP-1 macrophages were exposed to bacterial components of *H. pylori*, such as LPS and glycine extract (GE), which were obtained from *H. pylori* CCUG strain 17874, as described by Moran et al. [39] and Rechciński et al. [40], respectively. Transformation of macrophages into foam cells was visualized microscopically based on the presence of lipid droplets stained red by the Oil Red O method. Results presented in Figure 6A,B indicate that *H. pylori* components induced significant transformation of macrophages into foam cells. Moreover, among all the stimulators, the greatest foam-forming potential had *H. pylori* GE, containing mainly surface antigen proteins. As shown in Figure 6A, *H. pylori* GE was two times more effective in induction of foam cell formation than LPS *E. coli* (used in the experiment as a reference control) [41], and as efficient as 7-ketocholesterol (7-KCh), which served as the positive control of the transformation process. 7-KCh accumulates in oxidized lipoprotein deposits and it is known to be implicated in macrophage foam cell formation in atherosclerosis [42].

### 2.5. In Vitro Model of Vascular Endothelial Response Driven by H. pylori Components

**Figure 7:**
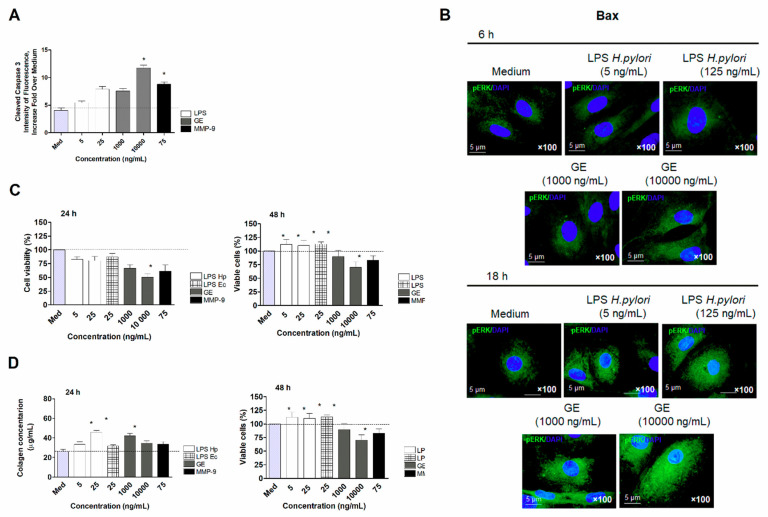
Detection of apoptosis intensity in vascular endothelial cells treated with *H. pylori* antigenic components: (GE) and lipopolysaccharide (LPS) or metalloproteinase (MMP)-9. (**A**) Cleavage of Caspase 3, presented as the intensity of the fluorescence fold increase across mediums. (**B**) Representative pictures of the expression of proapoptotic protein Bax in vascular endothelial cells analyzed with fluorescent microscopy at a 1000× magnification. (**C**) Endothelial cell viability tested in an MTT reduction assay. Results are presented as the average percentage of cells (control and stimulated with *H. pylori* antigens). (**D**) The concentration of Collagen I in culture media of vascular endothelial cells (control or stimulated with *H. pylori* antigens). Data represent the average values of four independent experiments performed in triplicates for each experimental variant. Statistical analysis was performed using the nonparametric Mann–Whitney U test with significance for * *p* < 0.05 obtained for unstimulated cells vs. cells treated with stimulators.

To investigate the effect of *H. pylori* components on dysregulation of vascular endothelial cell homeostasis, we developed an in vitro model of primary endothelial cells derived from aortic tissue of *Cavia porcellus* [27]. The in vitro model that we have developed is very feasible and mimics the in vivo situation that is taking place in the organism during infection with *H. pylori*. Thus, the primary endothelial cells derived from aortic tissue of *Cavia porcellus* were treated with *H. pylori* components: LPS or GE for 24 h, and then tested for the expression of cleaved form of Caspase 3 (CC3), the protein, which is the executioner of cell apoptosis. We showed that the *H. pylori* GE components upregulated apoptosis of primary vascular endothelial cells in a dose-dependent manner (Figure 7A). Importantly, *H. pylori* GE at the concentration of 10 µg/mL stimulated cleavage of Caspase 3 even more effectively than MMP-9, which served as the positive control [43]. Interestingly, *H. pylori* LPS showed a tendency to increase vascular endothelial cell apoptosis in a dose-dependent manner; however, the results were statistically insignificant (Figure 7A).

Next, we examined the involvement of pro-apoptotic Bax proteins in regulation cell apoptosis initiated by *H. pylori* antigenic components. Among several pro-apoptotic proteins, we picked Bax to visualize the expression in cells exposed to *H. pylori* LPS and GE for 6 and 18 h. As shown in Figure 7B, the expression of Bax in cells after 6 h of treatment was on the level of control regardless of the stimulator. Interestingly, after 18 h of cell exposure to *H. pylori* components, the expression of pro-apoptotic Bax increased—the most effectively for *H. pylori* GE. Our observation agreed with the data on expression of cleaved Caspase 3 (Figure 7A).

Moreover, we examined the metabolic activity of primary endothelial cells exposed to *H. pylori* components for 24 and 48 h using an MTT reduction assay. As shown in Figure 7C, 24-h exposure of vascular endothelial cells on *H. pylori* GE resulted in an almost 50% loss of cell viability. Interestingly, *H. pylori* LPS, as well as reference LPS *E. coli* caused only a slight, nonsignificant decrease in vascular endothelial cell viability, which correlated with the levels of cleaved Caspase 3 and Bax presented in Figure 7A,B, respectively. Continuation of the exposure of primary endothelial cells to *H. pylori* components up to 48 h caused a cell number increase in the milieu of *H. pylori* LPS, suggesting the ability of cells to recover over the control level—100% cell viability (Figure 7C). On the other hand, the number of vascular endothelial cells exposed to *H. pylori* GE remained significantly low, suggesting that cell apoptosis was irreversible. Finally, to investigate the role of *H. pylori* components in promoting vascular endothelial cell longevity/recovery, we measured the secretion of Collagen type I in the culture media. Our results presented in Figure 7D indicated that the concentration of Collagen I in the culture media of endothelial cells treated with *H. pylori* LPS for 24 h was significantly high and showed a tendency to increase continuously up to 48 h. On the contrary, reference LPS *E. coli* as well as MMP-9 did not support vascular endothelial cell secretion of the extracellular matrix. Interestingly, Collagen type I secretion by vascular endothelial cells stimulated with *H. pylori* GE was minor and noticeable only after 24h of cell exposure (Figure 7D)**.** Upregulation of Collagen I can be considered in terms of its proregenerative activity in response to the deleterious effects of *H. pylori* and/or components of the high-fat diet. However, overproduction of collagens by themselves or in correlation with complement activation may constitute a risk of the development and worsening of the atherosclerosis process [44].

**Figure 8:**
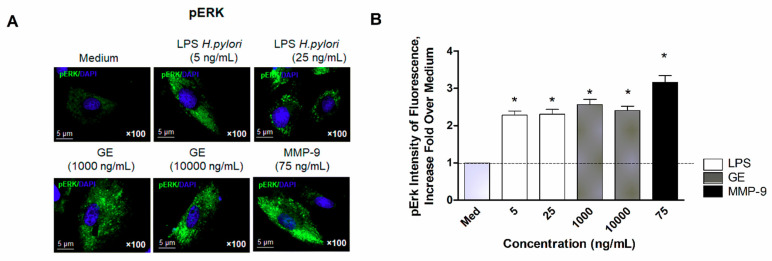
Analysis of ERK activation/phosphorylation (pERK) in vascular endothelial cells. (**A**) Representative pictures of pERK in endothelial cells unstimulated or treated with *H. pylori* antigenic components: glycine acid extract (GE), lipopolysaccharide (LPS) or metalloproteinase (MMP)-9 analyzed with fluorescent microscopy at magnification 1000×. (**B**) pERK expression, presented as the intensity of the fluorescence fold increase over the mediums. A minimum of 100 cells were analyzed. Statistical analysis was performed in the nonparametric U Mann-Whitney test with significance for * *p* < 0.05 obtained for unstimulated cells vs cells treated with stimulators.

Further, we investigated the expression of phosphorylated ERK (pERK) in vascular endothelial cells exposed to bacterial components of *H. pylori*. Microscopic analysis of expression of pERK in vascular endothelial cells (Figure 8A) indicated that *H. pylori* LPS as well as GE induced significant activation of this kinase protein, suggesting the initiation of ERK-dependent cell activation. Moreover, the analysis of fluorescence intensity (Figure 8B) revealed that all *H. pylori* components were almost as potent as MMP-9 in launching endothelial cell activation via the ERK pathway.

Finally, our initial study carried out on human HUVEC cells indicate the formation of a pro-inflammatory profile of these cells upon the stimulation with soluble *H. pylori* components (GE and LPS), detected as the upregulation of cell surface ICAM-1 adhesion molecules (data not shown). However, in the milieu of 7-kCh, which is the oxidized form of cholesterol, the expression of the surface level of ICAM-1 in HUVECs was lowered, which may suggest a regulatory activity of 7-kCh or the defense reaction of cells (data not shown). The mechanism of regulation of ICAM-1 expression by the *H. pylori* soluble components and 7-kCh should be further investigated. Recent studies have discovered that oxLDL may have both anti-inflammatory and pro-inflammatory properties, which can be explained based on the multiple LDL modification theory, which assumes that that LDL particles may undergo numerous modifications that change their size, density, and chemical properties during interaction with endothelial cells [45]. It has been shown that thioredoxinn-1, which regulates the redox balance, downregulates adhesion molecule expression induced by oxLDL, and due to this it can potentially participate in the protection against atherosclerosis [46].

## 3. Discussion

Because the occurrence of classic risk factors for development of atherosclerosis do not explain all cases of coronary heart disease (CHD), the concept that atherogenesis may have an infectious background is extremely possible. Since Mendall et al. (1994) [8] showed for the first time the elevation of specific antibodies towards *Helicobacter pylori* in patients with CHD, the evidence of these bacteria’s influence on the development of atherosclerosis should not been omitted [8]. Importantly, several studies supported the association between CagA^+^ *H. pylori* infection and CHD [47]. This observation was the result of research conducted in ethnic groups with a low incidence of classic risk factors for CHD and a high prevalence of *H. pylori* infection. Longo-Mbenza et al. (2012) [48] also showed that high levels of anti-*H. pylori* IgG were significantly associated with a higher risk of CHD in a group of Central Africans [48].

The Gram-negative bacterium *H. pylori* infects over a half of human population causing firstly an acute local inflammatory response followed by a persistent systemic inflammatory reaction. In this study, we used a unique model of experimental infection with *H. pylori* in *Caviae porcellus* previously developed and characterized in our laboratory by Walencka et al. (2015) [30]. To explore the link between *H. pylori* infection and hyperlipidemia in induction of an inflammatory response and initiation of a proinflammatory and proatherogenic environment, animals colonized with *H. pylori* were exposed to an experimental high-fat diet. The colonization of *H. pylori* locally in the stomach of infected animals was evaluated by histological analysis of gastric tissue sections (visualization of *Helicobacter*-like organisms—HLO) according to several principles of the Sydney system [30,35,36]. Histological verification was done by two independent histopathologists as a blind study by applying the grading system (range 0–3) for the presence of HLO. Even though the assessment of the *H. pylori* status by culture is included in the gold standard, it was not done for the paper, due to inaccuracy related to the existence of mixed flora in the guinea pig stomach [30]. On the other hand, detection of *H. pylori* antigens, which was done for the stool samples of animals, was selected as a good tool for the confirmation of *H. pylori* colonization [30].

Using an in vivo model of *H. pylori*-infected animals exposed to normal chow or high-fat diet, we measured the local and systemic soluble markers of inflammatory response, such as MPO, MMP-9 (the oxidative stress marker) and CRP, which is considered an indicator of coronary events [49,50]. Elevated CRP correlates with interleukin (IL)–6, tumor necrosis factor (TNF)–α, obesity or insulin resistance, which may indicate a link between chronic inflammation and endothelial dysfunction [51]. Our results indicate (Figure 3A,D) that the concentration of CRP in the serum and MPO in the gastric tissue was the highest in *H. pylori*-infected animals exposed to a high-fat diet, suggesting the synergistic effect of infectious and dietary risk factors for CHD in the process of development of a proatherogenic milieu. The main source of MPO in the gastric tissue are neutrophils infiltrating the gastric mucosa during *H. pylori* infection. We have shown previously the massive infiltration of neutrophils (Ly-6G positive cells) in the gastric tissue of *H. pylori*-infected guinea pigs [21]. Our results suggest (Figure 3A) that exposure of infected animals to a high-fat diet accelerates the process of neutrophils accumulation in the gastric tissue. The excess of activated neutrophils and release of MPO may result in the increase of *H. pylori*-induced injurious effects.

Neutrophils, if activated, also deliver MMP-9. Surprisingly, the level of MMP-9 (additional marker of CHD) in serum and gastric tissue of *H. pylori*-infected animals exposed to a high-fat diet was diminished (Figure 3B,C). The role of cholesterol in negative regulation of MMP-9 expression has been suggested by Kim et al. (2007) [52] and studied in normal human keratinocytes. MMP-9 can play a significant role in the tissue repair process by catalyzing the normal turnover of extracellular matrix (ECM) molecules and by regulating cell apoptosis and the cell cycle [53]. Negative regulation of MMP-9 expression by cholesterol support damage of the gastric epithelial barrier. As a consequence of such a scenario, the endothelium may get exposed to bacterial components of *H. pylori*, which may affect endothelial homeostasis. The relationship between MMP-9 and cholesterol metabolism was also confirmed using MMP-9-deficient mice (*Mmp9*^−/−^), which showed abnormal lipid gene transcriptional responses to dietary cholesterol supplementation [54]. The MMPs are regulated at different levels: gene expression, proteolytic activation of the proenzymes, inhibition of the catalytic activity by chemical and biological agents and complexing with specific tissue inhibitors (TIMPs). In animals infected with *H. pylori* receiving a high-fat diet, downregulation of MMP-9 could be regulated on different levels due to expansion of the adipose tissue and inflammatory response driven by *H. pylori*.

Our results indicate also that exposure of infected animals to a high-fat diet may downregulate the lymph node lymphocytes’ responsiveness to stimulation with *H. pylori* (Figure 2), even though we confirmed the presence of *H. pylori* bacteria in the gastric tissue (Table 1, Figure 1A,C). There are some studies on the effect of cholesterol on the immune response development. Thus, Mailer et al. (2017) [39] reported that hypercholesterolemia may facilitate proliferation of T lymphocytes and promote T-cell receptor (TCR) stimulation in CD4+ T cells but the study differed from ours substantially. Authors obtained CD4+ T lymphocytes from normal mice exposed to normal chow or cholesterol-containing diet and treated them with cholesterol only, so there was no infectious agent in the experimental set up. Moreover, Morey et al. (2018) [40] studied host cell response to *H. pylori* infection in the cholesterol accompaniment. The authors showed that *H. pylori* can bind to cholesterol causing cholesterol depletion within cell lipid rafts, which may influence cellular cytokine receptors assembly and result in blocking of the JAK/STAT signaling pathway.

Guinea pigs are considered a good model for studying the lipid metabolism, not only because of the human-like architecture of the aortic wall [55] but due to the resemblance of the mechanisms of initiation and progression of atherosclerosis in response to a high-fat diet [56]. Moreover, rodent models, including guinea pigs, are increasingly employed to study the development of arterial stiffness and foam cell formation, which accompany atherosclerosis [33,34,57]. We introduced photoplethysmography to investigate the elasticity of animals’ arteries, which can be diminished due to an inflammatory response and atherogenesis. Our data indicate that the arteries of infected animals, regardless of diet, were the least elastic, suggesting the prominent role of the infectious agent in arterial stiffening (Figure 4). Our results agree with Choi et al. (2019) [58], who demonstrated that *H. pylori* seropositivity is related to increased arterial stiffness and this correlation was more noticeable in elderly individuals [58]. Saijo et al. (2005) [59] also confirmed a link between *H. pylori* seropositivity and aortic stiffness in Japanese individuals [59].

Our results showed no presence of atherosclerotic plaques in the animal aorta; however, we showed the infiltration of inflammatory cells into the internal wall of the endothelium in response to chemotactic signals (Figure 5A,B). This may provide conditions for direct interactions between these two types of cells. Our initial study using the in vitro model of HUVEC cells showed the upregulation of IL-8 production in the milieu of *H. pylori* components (data not shown). Formation of atherosclerotic lesions is associated with the increased infiltration and reactivity of immune cells [60], particularly monocytes and macrophages, which transform into foam cells upon ingestion of oxLDL. Activated macrophages and other inflammatory cells release chemokines that stimulate the migration of smooth muscle cells, which together with foam cells, and form a fibrous cap. Finally, foam cells undergo apoptosis and together with cholesterol crystals form lipid plaque cover [61]. These processes are facilitated by interferon gamma (IFN–γ) and tumor necrosis factor alpha (TNF–α) secreted by T helper (Th)-1 lymphocytes, as well interleukin -12 (IL–12) produced by macrophages and foam cells [62]. We did not perform immunohistochemical staining to differentiate macrophages among immunocompetent cells adhering to the vascular endothelium, nor the detection of foam cells, because the assay is based on the chemical staining of lipid bodies present in the cell cytoplasm, and therefore is limited at the sensitivity level. To explore whether *H. pylori* components can induce the transformation of monocytes/macrophages into foam cells, we used an in vitro model of THP-1 cells, as previously described [41,42,63]. Results presented in Figure 6A,B indicated that *H. pylori* components and especially glycine extract revealed strong foam-forming potential comparable with 7-KCh, which served as a positive control of the transformation process [24]. We exposed THP-1 macrophages to soluble *H. pylori* antigens to mimic the situation that is taking place during the infection. Macrophages, which are involved in the process of plaque formation, are exposed to soluble bacterial antigens of *H. pylori*, which are getting into the circulation because of gastric epithelial cell barrier disruption [19,20,21,22]. Moreover, we have shown previously [5,21] that soluble *H. pylori* antigens can induce oxidative stress in cells, which is followed by lipid peroxidation, shown as the increase of 4-hydroxy-2-nonenal (4HNE). Our results refer to Li et al. (2000) [64], who discussed the role of infectious agents, including *H. pylori*, in inducing the transformation of macrophages into foam cells in a TLR-2- and TLR-4-dependent way [64].

Endothelium plays a major role in maintaining vascular homeostasis; therefore, when it is damaged loses functional integrity and becomes dysfunctional. Endothelial dysfunction usually leads to an increase in tension, vascular wall remodeling, vascular inflammation, platelet adhesion and aggregation. These processes contribute to the development of atherosclerosis [2]. To evaluate the potential contribution of *H. pylori* components on induction of the proatherogenic endothelial cell environment, we employed an in vitro model of vascular endothelial cells derived from aortic tissue of *Cavia porcellus*. Our data indicated that *H. pylori* GE activated vascular endothelial cells in an ERK-dependent way (Figure 8A,B), and initiated cell apoptosis—the expression of cleaved Caspase 3 (Figure 7A) and Bax protein (Figure 7B) as well as lowered cell viability (Figure 7C). Interestingly, we have observed that vascular endothelial cells exposed to *H. pylori* LPS rebuild viability (over 100%) after 48 h of treatment (Figure 7C), and this phenomenon was parallel with release of Collagen I into the culture medium (Figure 7D). Wang et al. (2016) [65] described the role of CagA *H. pylori* in apoptosis regulation of rat glomerular mesangial cell by promoting proliferation and secretion of Collagen I [65]. The importance of Collagen production by endothelial cell was also discussed by Kusuma et al. (2012) [66] and Davis et al. (2005) [67], who highlighted the crucial role of it in maintaining the integrity of mature blood vessels and promoting angiogenesis. Li et al. (2019) [68] showed that bacterial LPS can activate Toll-like receptor (TLR)-4 signaling, leading to the overexpression of Collagen I, via increased production of transforming growth factor (TGF)-β. Consequently, increased expression of Collagen I caused the induction of hypertrophic skin lesions and scar formation [68]. Moreover, it has been shown by Shields et al. (2011) [69] that complement components C3 and C4 bind to collagen and elastin in the vascular wall, causing an increase in vascular stiffness and atherosclerosis development [69]. Thus, the potentially excessive bacterial LPS-driven stimulation of collagen production at the site of endothelial dysfunction in atherosclerosis may lead to the augmentation of the complement-dependent inflammatory response. In this context, *H. pylori* LPS as well as CagA, by stimulating collagen overproduction, may contribute to the development of atherogenesis.

Summarizing, the in vivo model of *H. pylori* infection accompanied with a high-fat diet gave us an opportunity to investigate the role of infectious agents and dietary components, acting separate and in cooperation, in the development of a proatherogenic endothelial cell environment. Oxidative stress increased during *H. pylori* infection can be a perfect milieu for lipids to get oxidized. In such conditions, pro-atherogenic 7-kCh and aldehydes, including 4-hydroxy-2-nonenal, are formed to react with proteins and cause significant functional alterations affecting signaling pathways and inducing cell membrane damage [70]. Our in vitro study using vascular endothelial primary cells derived from *Cavia porcellus* also allowed us to explore the proinflammatory influence of the soluble *H. pylori* components on ERK-dependent activity and lifespan regulation.

Further investigation needs to be done especially in the context of Collagen I activation in response to *H. pylori* stimulation. Importantly, collagens are highly vulnerable to proteolytic digestion, remodeling and mineralization—processes that may drive atherosclerosis [44].

## 4. Materials and Methods

### 4.1. Animal Studies

All studies involving animals were approved by the Local Ethics Committee (LKE9) for Animal Experiments of the Medical University of Lodz, Poland, which was established by the Ministry of Science and Higher Education in Poland (Decision 44/LB105/2018). Ethics Statement in vivo experiments were developed according to the EU directive (Directive 2010/63/EU of the European Parliament and of the Council of 22 September 2010 on the protection of animals used for scientific purposes (Dz.U. L 276 z 20.10.2010, s. 33–79)).

### 4.2. H. pylori Infection in Caviae porcellus (Guinea Pigs)

Adult (three-month-old) male and female Himalayan guinea pigs (400–600 g), free of pathogens, were housed in the Animal House at the Faculty of Biology and Environmental Protection, University of Lodz (Lodz, Poland), kept in cages with free access to drinking water and fed with standard chow or a high-fat diet. The special experimental food for guinea pigs contained 19.5% fat, 15% sucrose and 0.35% cholesterol. The crude nutrients expressed as percentage contained proteins (17.5%), fat (19.5%), fiber (11.0%), ash (6.7%), starch (8.1%) and sugar (17.5%), together with some nutritional additives, such as Vitamin A, Vitamin D_3_, Vitamin E, Vitamin K_3_, Vitamin C, iron, zinc, manganese, copper, selenium and calcium (Vivari, Warszawa, Poland). The animals were inoculated per os with *Brucella* broth or *H. pylori* reference strain CCUG 17874 (positive for VacA and CagA), obtained from the Culture Collection, University of Gothenburg (Gothenburg, Sweden), as previously described [29,30]. The animals were monitored daily (body weight, water and food intake, behavioral symptoms, skin and fur condition, diarrhea) until Day 60. The animals were euthanized on Day 7, 28 and 60 with an overdose of sodium barbiturate (Morbital, Biowet, Puławy, Poland). The gastric tissue, heart and aorta were collected for histopathology, whereas blood samples were processed to obtain serum. *H. pylori* infection in the guinea pigs was confirmed by visualization of *Helicobacter*-like organisms (HLO) in thin layer sections of the stomach tissue, which were stained previously with a Giemsa stain solution and analyzed according to the Sydney scale using light microscopy, as previously described by us [29]. The preparations were assessed by two independent histopathologists (Cytopath Poland). Additionally, anti-*H. pylori* IgG antibodies in the serum samples were detected by the laboratory enzyme linked immunosorbent assay (ELISA), as previously described by Rechcinski et al. (1997) [40]. During active *H. pylori* infection, antigens of these bacteria are discharged into the intestine. The presence of *H. pylori* antigens in the feces is evidence of an active infection, and the concentration of *H. pylori* antigens in the stool samples of infected animals was evaluated by commercial ELISA assay as previously described by Walencka et al. (2015) [30] (Immunodiagnostic AG, Bensheim, Germany).

### 4.3. ELISA Assays: MMP-9, CRP

The concentration of total MMP-9 in animal serum samples and gastric tissue homogenates was measured using commercial ELISA (MyBiosource, San Diego, CA, USA), as recommended by the manufacturer (sensitivity 0.1 ng/mL). The concentration of CRP in serum samples was detected using commercial ELISA test (Cloude-Clone Corporation, TX, USA; sensitivity 0.062 ng/mL). Absorbance was measured using Victor 2 reader (Wallac, Oy, Turku, Finland) at a wavelength of 450 nm.

### 4.4. Determination of Oxidative Stress (MPO)

Myeloperoxidase was detected in gastric tissue homogenates as described by Gonciarz et al. (2019) [21]. Briefly, 75 µL/well of gastric tissue homogenate was mixed with 150 µL/well of substrate—3,3′5,5′tetrametylobenzydyne—TMB (20 mM TMB/DMSO in NaH2PO4 buffer pH 5.4 with addition of 30% H_2_O_2_). The colorimetric reaction was stopped with 1M H2SO4. Absorbance was measured using Victor 2 reader (Wallac, Oy, Turku, Finland) at a wavelength of 450 nm.

### 4.5. Cell Proliferation

Total mesenteric lymph node lymphocytes were isolated as routinely done in the laboratory [29] and then subjected to the proliferation assay based on the thymidine incorporation method as described in our previous study by Miszczyk et al. (2014) [29]. The results were expressed as the mean cpm/culture ± standard deviation (SD). The stimulation index (SI), expressing the relative cpm ratio, was calculated by dividing the cpm counts/min, obtained for the cell cultures with a stimulant, by the cpm counts/min for the cell cultures without a stimulant. The SI values greater than or equal to 1.5 were considered as a positive result in the proliferation assay.

### 4.6. Measurement of Arterial Stiffness

In vivo vascular elasticity was determined for animals all groups by measuring photoplethysmography (Indus Instruments, Houston, TX, USA) at the right hind limb artery location (Day 60 of the experiment). Before the procedure, the animals had unlimited access to water and food as well as a body temperature in the normal range. Briefly, animals were positioned supine on a temperature-controlled board (THM100, Indus Instruments) and kept under anesthesia (1% isoflurane) during the measurement. An infrared sensor was fixed over the artery of the right hind limb. After pulse rate stabilization (approx. 20 s) the amplitudes were recorded. The examination lasted minimally 15 s to obtain at least 20 good quality waves. Extremely high and low values of the amplitudes were excluded from each analysis. At last, only one average amplitude value was chosen by the researcher as the representative data. The mean values of the amplitudes (AU) were compared between groups of animals.

### 4.7. Cell Culture

Primary *Caviae porcellus* vascular endothelial cells were derived from segments of aortic tissue isolated from normal animals (guinea pigs) as previously described by Wang JM. et al. (2017) [71]. Human umbilical vein endothelial cells of the HUVEC line were obtained commercially (C2517A, Lonza, Walkersville, MD, USA). Cells were cultured in endothelial growth medium (EGM-2) enriched with fetal bovine serum (FBS), hydrocortisone, fibroblast growth factor (fFGF), vascular endothelial growth actor (VEGF), Epidermal Growth Factor (EGF), ascorbic acid, gentamicin sulphate, amphotericin (GA-1000) and heparin (Lonza, Poland), at 37 °C in a humidified atmosphere containing 5% CO_2_. Every 2–3 days, the medium was changed, and the cells were passaged at 80–90% confluence. Cells were trypsinized, washed with phosphate buffered saline (PBS), and their viability was then assessed by trypan blue staining and cell suspensions were adjusted to working density.

### 4.8. Bacterial Stimuli

Glycine acid extract (GE) containing surface antigens from the reference *H. pylori* strain CCUG 17874 was obtained as previously described by Rechciński et al. (1997) [40]. The protein composition was assessed by us previously by sodium dodecyl sulphate—polyacrylamide gel electrophoresis (SDS-PAGE) and Western blotting using reference serum samples from patients infected with *H. pylori* [17,72,73]. The major proteins identified in *H. pylori* GE were 120 kDa (CagA), 87 kDa (VacA), 66 kDa (UreB), 60 kDa (Hsp), 29 kDa (UreA) and 22–26 kDa. The GE contained <0.001EU/mL of LPS, as shown by the chromogenic *Limulus* amebocyte lysate test (Lonza, Belgium), and was used for in vitro experiments at the concentration of 1 and 10 µg/mL. LPS from the reference *H. pylori* CCUG 17874 strain was obtained by hot phenol-water extraction and purified by proteinase K, DNase and RNase treatment as previously described [39]. *H. pylori* LPS was used for in vitro experiments at the concentration of 5 and 25 ng/mL, and LPS *E. coli* (serotype O55:B5, Sigma-Aldrich) at the concentration of 25 ng/mL. Active human recombinant MMP-9 (BioVision, CA, USA) was used for in vitro experiments at the concentration of 75 µg/mL, whereas 7KCh (Sigma-Aldrich) 40 ng/mL. The concentration stimulators were adjusted experimentally or adopted from previously performed experiments [27,74,75].

### 4.9. Foam Cell Formation

Human monocyte leukemic cell line (THP-1) was purchased from (ATCC, UK). Nonadherent cells were cultured in RPMI-1640 medium supplemented with 10% heat-inactivated FBS (Biowest, France), 2 mM l-glutamine and 100 U/mL streptomycin-penicillin (Biowest, France) at 37 °C with 5% CO_2_. Foam cell formation assay was conducted as described by Khan et al. (2014) [41]. Briefly, THP-1 cells plated at 3 × 10^5^ cells/well on glass coverslips (placed in 12 well plate) were differentiated into macrophages with 50 nM phorbol-12- myristate-13-acetate (PMA; Sigma-Aldrich) for 72 h at 37 °C with 5% CO_2_. After 72 h, the adherent macrophages were exposed to *H. pylori* GE (10 ug/mL, 1 ug/mL, 0.1 ug/mL), *H. pylori* LPS (25 ng/mL and 5 ng/mL), LPS *E. coli* (25 ng/mL) and 7-KCh (conc. 40 ng/mL) for 24 h at 37 °C with 5% CO_2_. After 24 h, the cells were fixed with 4% paraformaldehyde in PBS for 15 min, and covered with Oil Red O (Sigma) at a working solution (0.6% (*m*/*v*) in a 60% (*v*/*v*) isopropanol solution for 30 min. Next, the cells were counterstained with a hematoxylin solution (Elektro Med, Niepolomice, Poland) for 3 min. Stained cells were visualized with light microscopy (Eclipse 50i, Nikon; 1000× magnification) with a photo camera, a Power Shot A 640 (Canon), integrated. Lipid droplets appeared red and nuclei appeared blue.

### 4.10. Cell Metabolic Activity/Viability Assay

Metabolic activity of primary vascular endothelial cells treated with *H. pylori* components, LPS *E. coli* and MMP-9 was evaluated colorimetrically using thetetrazolium yellow dye MTT (3-(4,5-dimethylthiazol-2-yl)-2,5-diphenyltetrazolium bromide), which was reduced by living cells to soluble purple formazan crystals, as previously described [20]. Absorbance at 570 nm was estimated with a Victor 2 plate reader (Wallac, Oy, Turku, Finland). The results were presented as the median percentage with the range, relative to untreated cells. The effectiveness of MTT reduction was calculated based on Equation (1):

(1) $$\begin{array}{ll} MTT\;reduction\; & relative\;to\;untreated\;cells\;\left(\%\right)\\ & =\;(absorbance\;of\;treated\;cells\\ & /absorbance\;of\;untreated\;cells\;\times \;100\%)\;-\;100\% \end{array}$$

### 4.11. Extracellular Matrix Production (Collagen I Concentration)

The concentration of Collagen I was detected as described by Wang et al. (2016) [65]. Briefly, the level of Collagen I was measured in culture media of primary vascular endothelial cells treated with *H. pylori* components, LPS *E. coli* and MMP-9, for 24 and 48 h, using the pair of antibodies: primary anti-Collagen 1 polyclonal antibodies and horseradish peroxidase (HRP) conjugated anti-Collagen I polyclonal secondary antibodies (Bioss Antibodies, MA, USA).

### 4.12. Cell Apoptosis

Apoptosis of primary vascular endothelial cells was detected as routinely done in the laboratory [22]. Briefly, after 24 h of stimulation with *H. pylori* components, MMP-9 cells were fixed with 4% formaldehyde, permeabilized with 0.2% Triton X-100 in PBS and incubated with primary anti-cleaved Caspase 3 antibodies (Cell Signaling Technology, Danvers, MA, USA), followed by incubation with fluorescein isothiocyanate (FITC)-conjugated secondary antibodies (Invitrogen Carlsbad, CA, USA). Cell nuclei were counterstained with 4′,6-diamidino-2-phenylindole (DAPI) (Sigma-Aldrich, Saint Louis, MI, USA). In some experiments, the level of pro-apoptotic Bax protein was detected in cells treated with stimulators for 6 and 18 h with anti-Bax antibodies (Cell Signaling Technology, Danvers, MA, USA). Stained cells were visualized in fluorescent microscope at appropriate wavelengths: FITC (excitation 495 nm, emission 519 nm) and DAPI (excitation 358 nm, emission 461 nm) at 1000× magnification (Zeiss, Axio Scope, A1, Jena, Germany) Fluorescence intensity was measured using ImageJ software. Three independent experiments were carried out in three replicates for each experiment.

### 4.13. Erk Activation/Phosphorylation (pErk)

Primary vascular endothelial cells were cultured on glass inserts placed in 6-well culture plate (Thermo Fisher Scientific, Waltam, MA, USA) at a density of 5 × 10^5^ cells/well (volume 0.5 mL) in EGM-2 complete medium (37 °C, 5% CO_2_). After 24h of cell exposure on *H. pylori* GE (10 ug/mL and 1 ug/mL), *H. pylori* LPS (25 ng/mL and 1 ng/mL) and MMP-9 (75ug/mL) cells were fixed with 4% formaldehyde solution for 20 min, washed 3 times with PBS, followed by incubation with 0.02% TritonX-100 for 10 min to permeabilize the membranes. Next, cells were incubated with 5% bovine serum albumin (BSA) in phosphate buffered saline (PBS) to block unspecific binding and then with primary rabbit anti-phospho-Erk 1/2 (Thr202/Tyr204) antibody (Cell Signaling Technology, Danvers, MA, USA) followed by FITC-conjugated chicken anti-rabbit secondary antibody (Thermo Fisher Scientific, Waltam, MA, USA). Activation of Erk was visualized using fluorescence microscope (Zeiss, AxioScope, A1, Jena, Germany) at a wavelength of 550 nm (excitation) and 590 nm (emission).

### 4.14. Statistical Analysis

All values are expressed as the median values with a range. The statistical significance in individual experiments was tested using Statistica 12 PL software using the nonparametric Mann–Whitney U test or Kruskal–Wallis test. Results were considered statistically significant when * *p* < 0.05, ** *p* < 0.01, and *** *p* < 0.001.

## Figures and Tables

**Table 1:** Validation of *H. pylori* infection by grading *Helicobacter*-like organisms (HLO) and gastritis in the stomach of guinea pigs inoculated with the reference *H. pylori* strains at 7, 28 and 60 days of infection. The visual grading system for HLO is 0—no bacteria detected in gastric crypts; 1—mild level of colonization (some bacteria detected in gastric crypts); 2—moderate level of colonization (bacteria detected in most gastric crypts); and 3—severe level of colonization (bacteria present in all gastric crypts). For cell infiltration it is 0—no infiltration of inflammatory cells; 1—moderate infiltration of inflammatory cells; and 2—increased infiltration of cells.

Sample Diagnostic Assay		*H. pylori* CCUG 17874			*Brucella* Broth	*H. pylori* CCUG 17874			*Brucella* Broth
		Normal Diet				High Fat Diet			
		7 Days Post Infection (*n* = 5)	28 Days Post Infection (*n* = 5)	60 Days Post Infection (*n* = 5)	Control (*n* = 5)	7 Days Post Infection (*n* = 5)	28 Days Post Infection (*n* = 5)	60 Days Post Infection (*n* = 5)	Control (*n* = 5)
Histopathology									
Gastric tissue	HLO grading:								
	-Giemsa	1 1 1 2 1	1 2 1 2 2	1 2 2 1 0	0 0 0 0 1	1 1 1 1 2	1 2 2 1 1	2 1 2 2 0	0 0 0 0 0
	Immune cell grading (H&E)								
	-granulocytes	1 1 1 1 1	1 2 1 2 1	2 1 2 2 2	0 0 0 0 0	1 1 1 1 1	1 2 1 2 2	2 2 2 2 2	0 0 0 0 0
	-lymphocytes	1 1 1 2 1	1 2 2 2 2	2 1 2 2 1	0 0 0 0 0	1 1 1 1 1	2 1 2 2 2	1 2 2 1 2	0 0 0 0 0

## Abbreviations

4HNE	4 hydroxynonenal
7-kCh	7-ketocholesterol
BSA	Bovine serum albumin
CagA	Cytotoxin associated gene A protein
CC3	Cleaved Caspase 3
CHD	Coronary Heart Disease
CRP	C-reactive protein
DAPI	4’,6-diamidino-2-phenylindole
EGF	Epidermal growth factor
EGM-2	Endothelial growth medium
ELISA	Enzyme-linked immunosorbent assay
FBS	Enriched with fetal bovine serum
FGF	Hydrocortisone, fibroblast growth factor
FITC	Fluorescein isothiocyanate
GE	Glycine acid extract
HLO	*Helicobacter*-like organisms
Hsp	Heat shock protein
HUVEC	Human umbilical vein endothelial cells
ICAM-1	Intracellular adhesion molecules 1
ICAM-2	Intracellular adhesion molecules 2
IL-6	Interleukin
LDL	Low-density lipoproteins
LPS	Lipopolysaccharide
MCP-1	Macrophage chemotactic protein-1
MMP-9	Metalloproteinase
MPO	Myeloperoxidase
NF-κB	Nuclear factor kappa B
oxLDL	Oxidized low-density lipoproteins
PBS	Phosphate buffered saline
PECAM	1—platelet endothelial cell adhesion molecule 1
pERK	Phosphorylated extracellular signal-regulated kinase
PHA	Phytohemagglutinin
SDS-PAGE	Sodium dodecyl sulphate—polyacrylamide gel electrophoresis
TCR	T-cell receptor
TGF-β	Transforming growth factor
THP-1	Human monocyte leukemic cell line
TLR-4	Toll-like receptor 4
TMB	3,3′5,5′tetrametylobenzydyne
TNF–α	Tumor necrosis factor
UreA	Urease subunit A
UreB	Urease subunit B
VacA	Vacuolating cytoxin A
VCAM-1	Vascular cell adhesion molecule 1
VEGF	Vascular endothelial growth actor
